# Análise Transcriptômica por Sequenciamento de Alto Rendimento da Expressão de lncRNAs e mRNAs em Pacientes com Fluxo Lento Coronário

**DOI:** 10.36660/abc.20240847

**Published:** 2025-08-22

**Authors:** Haibing Jiang, Yi Yang, Xueqin Zhai, Lijing Zhang, Aerziya Kahaerjiang

**Affiliations:** 1 Hospital of Traditional Chinese Medicine Xinjiang Medical University Urumqi China Hospital of Traditional Chinese Medicine Affiliated to Xinjiang Medical University, Urumqi– China; 2 Xin Jiang Medical University Urumqi Xinjiang China Xin Jiang Medical University, Urumqi, Xinjiang – China

**Keywords:** Sequenciamento de Nucleotídeos em Larga Escala, RNA Mensageiro, RNA Longo não Codificante, Inflamação

## Abstract

**Fundamento:**

Os RNAs longos não codificantes (lncRNAs) e os microRNAs (miRNAs) são considerados elementos-chave na fisiopatologia do fluxo lento coronário (FLC).

**Objetivos:**

Este estudo teve como objetivo explorar as redes biológicas complexas envolvidas no FLC por meio do sequenciamento do transcriptoma completo, visando identificar potenciais biomarcadores diagnósticos e alvos terapêuticos.

**Métodos:**

O sequenciamento do transcriptoma completo foi realizado em amostras de três pacientes com FLC e três indivíduos controle pareados. Foi considerado estatisticamente significativo o valor de p < 0,05.

**Resultados:**

Foram identificados 854 lncRNAs diferencialmente expressos, sendo 425 com expressão reduzida e 429 com expressão aumentada. A análise de vias do KEGG mostrou enriquecimento significativo de lncRNAs em vias associadas a doenças cardiovasculares, distúrbios endócrinos e metabólicos, e progressão de doenças neurodegenerativas. Além disso, foram identificados 1.999 mRNAs diferencialmente expressos, dos quais 990 estavam regulados negativamente e 1.009, positivamente. A análise de função molecular revelou participação em ligação a proteínas, regulação da atividade de quinases, atividade de transferase de ubiquitina-proteína e ligação a RNA. A análise de vias KEGG indicou que os mRNAs diferencialmente expressos estavam principalmente envolvidos em autofagia, sarampo, proteólise mediada por ubiquitina, via de sinalização por receptores do tipo NOD, via de sinalização do fator de necrose tumoral (TNF), via de sinalização por receptores do tipo Toll (TLR) e via de sinalização NF-κB.

**Conclusões:**

Os mRNAs diferencialmente expressos apresentaram enriquecimento significativo em vias KEGG relacionadas à autofagia, sarampo e degradação mediada por ubiquitina, além de cascatas de sinalização envolvendo receptores do tipo NOD, TNF, TLR e NF-κB. Novos estudos são necessários para validar esses achados.

## Graphical abstract

**Figura Central:**
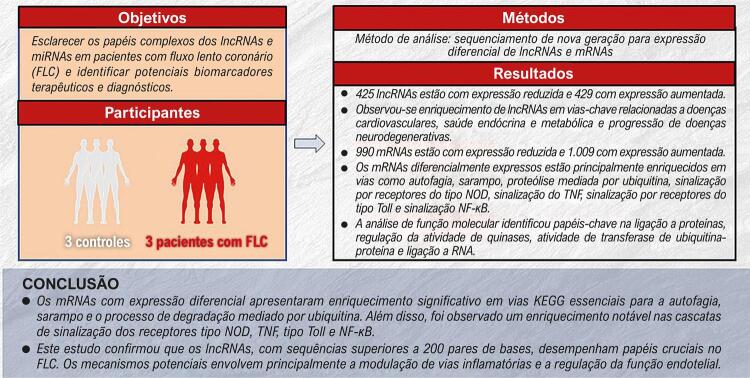
Análise Transcriptômica por Sequenciamento de Alto Rendimento da Expressão de lncRNAs e mRNAs em Pacientes com Fluxo Lento Coronário

## Introdução

O fluxo lento coronário (FLC) está intimamente associado ao surgimento de diversas manifestações clínicas do fenômeno do FLC.^1-5^ Essa condição tem sido relacionada a diversos fatores contribuintes, incluindo transtornos psiquiátricos, níveis séricos de salusina-β, homocisteína, cistatina C, índice de massa corporal e inflamação imune sistêmica.^2,3,6,7^ A resposta inflamatória pode desempenhar um papel central em múltiplos processos fisiopatológicos, como a obstrução microvascular e a formação de microtrombos, considerados elementos-chave na patogênese da disfunção da microcirculação coronariana.^8-11^

Estudos recentes demonstraram que determinados RNAs longos não codificantes (lncRNAs) podem regular fatores inflamatórios a jusante, influenciando assim o desenvolvimento do FLC.^9,12^ Danaii et al. propuseram que os microRNAs (miRNAs) podem atuar como potenciais biomarcadores para o diagnóstico do FLC e o monitoramento da progressão da doença arterial coronariana (DAC) em pacientes acometidos.^13^ Com base nesses achados, os lncRNAs e miRNAs parecem desempenhar papéis significativos na fisiopatologia do FLC. No entanto, considerando a complexidade da condição e as diversas funções dos RNAs não codificantes no sistema cardiovascular, focar em apenas algumas moléculas pode não proporcionar uma compreensão abrangente dos mecanismos subjacentes.

A análise do transcriptoma completo oferece uma perspectiva mais ampla, permitindo a identificação dos principais lncRNAs e miRNAs envolvidos no FLC, bem como suas possíveis interações. Essa abordagem pode contribuir para uma compreensão mais profunda da base molecular do FLC e apoiar futuras aplicações clínicas.

O sequenciamento de RNA (RNA-seq) tem sido amplamente utilizado para investigar perfis de expressão gênica em tecidos e células cardíacas, oferecendo subsídios para a compreensão das alterações moleculares associadas a condições como o FLC e a síndrome de Takotsubo, uma forma de doença microvascular coronariana.^14,15^ Um estudo transcriptômico do FLC, utilizando RNA extraído de monócitos do sangue periférico, revelou padrões diferenciais de expressão gênica e uma associação com a inflamação.^16^ No entanto, a patogênese do FLC permanece complexa e pouco compreendida, e até o momento apenas alguns estudos aplicaram o sequenciamento do transcriptoma especificamente ao FLC.

Dessa forma, o presente estudo teve como objetivo investigar o panorama transcriptômico de pacientes com FLC por meio do sequenciamento de alto rendimento de leucócitos do sangue periférico, a fim de identificar genes e vias potencialmente envolvidas no desenvolvimento do FLC.

## Métodos

### Participantes

Este estudo, apoiado pelo financiamento nº 2016XE0113, foi conduzido de acordo com os princípios éticos estabelecidos na Declaração de Helsinque. Todos os participantes foram plenamente informados sobre os objetivos do estudo e assinaram o termo de consentimento livre e esclarecido. O protocolo do estudo foi aprovado pelo Comitê de Ética em Pesquisa, garantindo a proteção dos direitos e do bem-estar dos participantes.

Foram incluídos três pacientes diagnosticados com FLC com base em angiografia coronariana, além de três indivíduos com achados coronarianos normais nesse exame, recrutados no Hospital de Medicina Tradicional Chinesa da Região Autônoma Uigur de Xinjiang. Esses indivíduos foram alocados, respectivamente, nos grupos caso e controle. Todos os participantes foram inicialmente suspeitos de apresentar DAC não complicada e não possuíam histórico de diabetes, miocardiopatia, vasculite, doenças do tecido conjuntivo ou doenças endócrinas.

### Critérios diagnósticos

O FLC foi definido como a perfusão tardia de qualquer vaso coronário distal principal, excedendo três ciclos cardíacos, conforme observado durante a angiografia coronariana.

### Critérios de exclusão

Os participantes foram selecionados com base em critérios rigorosos de inclusão e exclusão, a fim de garantir a confiabilidade e validade dos achados do estudo. Foram excluídos pacientes que apresentavam hipotensão (pressão arterial sistólica ≤ 90 mmHg no momento da angiografia) na ausência de uso de medicamentos vasodilatadores, ou frequência cardíaca < 60 batimentos por minuto sem influência de fármacos que modulam a frequência cardíaca. Também foram excluídos indivíduos que haviam apresentado infarto agudo do miocárdio ou síndrome coronariana aguda — incluindo angina instável — no mês anterior. Além disso, pacientes com qualquer estenose de artéria coronária > 50% não foram considerados elegíveis para inclusão. Dados clínicos, índices laboratoriais e amostras de sangue foram coletados e analisados de todos os participantes.

### Métodos experimentais

#### Construção do banco de dados e processo de sequenciamento

O RNA total foi extraído das amostras de sangue após centrifugação, lise e precipitação. A integridade do RNA isolado foi avaliada por meio de procedimentos analíticos padrão, seguida por uma análise de qualidade. Em seguida, os RNAs ribossômicos (rRNAs) foram removidos para o enriquecimento de mRNAs e RNAs não codificantes. O RNA restante foi fragmentado e transcrito reversamente em DNA complementar (cDNA). Em seguida, os cDNAs com extremidades cegas passaram por ligação de adaptadores e adição de caudas. A biblioteca de cDNA resultante foi submetida à separação por eletroforese em gel de agarose, sendo recortada a faixa correspondente ao tamanho-alvo. Os fragmentos de cDNA selecionados foram então amplificados por reação em cadeia da polimerase (PCR) e submetidos ao sequenciamento de alto rendimento utilizando uma plataforma automatizada.

#### Coleta de amostras e processamento de sangue total

A etapa inicial consistiu no registro e identificação precisos de amostras de 4 ml de sangue total coletadas de pacientes diagnosticados, com cada amostra rotulada de acordo com o prontuário do paciente. Após a centrifugação, os componentes plasmáticos e celulares foram separados. O plasma, localizado na camada superior após a centrifugação, foi transferido para tubos Eppendorf de 1,5 ml, sendo adicionados 400 μl de plasma em cada tubo. Os tubos foram então selados e armazenados em ultrafreezer a –80 °C para preservação.

A centrifugação foi realizada a 3.000 rpm durante 10 minutos, garantindo a separação eficiente do plasma. O pellet celular resultante foi ressuspenso em Tampão de Lise ACK (amônio-cloreto-potássio) (Bioseth, Zhenjiang, China) e transferido para tubos Eppendorf novos de 1,5 ml. O pellet de cada paciente foi dividido igualmente em dois grupos para análises posteriores, assegurando a integridade das amostras para os procedimentos subsequentes.

Os tubos Eppendorf contendo as células ressuspensas foram incubados à temperatura ambiente por 5 minutos para promover a lise celular. Em seguida, foi realizada a centrifugação para separação dos componentes. Para melhorar a eficiência da lise, o tratamento com Tampão de Lise ACK foi repetido duas a três vezes, garantindo a ruptura completa das células. Após a etapa final de lise, as amostras foram lavadas com solução salina fisiológica para remover resíduos do tampão.

Para a extração de RNA, os leucócitos de um dos conjuntos de tubos Eppendorf de 1,5 ml foram processados utilizando um protocolo de ressuspensão e precipitação à base de TRIzol, com o reagente TRIzol (Invitrogen, EUA). Outro conjunto de tubos contendo leucócitos foi mantido sem tratamento e armazenado a –80 °C para uso futuro.

#### Teste das amostras de RNA

A integridade e a pureza das amostras de RNA foram avaliadas por meio de um processo de controle de qualidade (CQ) em múltiplas etapas. Inicialmente, foi realizada eletroforese em gel de agarose para detectar degradação e contaminação. Esse método separa fragmentos de ácidos nucleicos por tamanho, permitindo uma avaliação visual da integridade do RNA. Em seguida, foi realizada análise espectrofotométrica para determinar a pureza do RNA, utilizando-se a razão A260/A280 como indicador padrão. A quantificação de alta precisão da concentração de RNA foi então realizada com o fluorímetro Qubit® 3.0, assegurando uma medição precisa do rendimento de RNA. Por fim, foi utilizado o sistema Agilent 2100 Bioanalyzer com o kit RNA Nano 6000 Assay para avaliação da integridade do RNA. Essa análise gerou um número de integridade de RNA (RIN), que fornece uma medida quantitativa da qualidade do RNA.

#### Construção e teste da biblioteca

Para a construção da biblioteca de lncRNAs, foi selecionada uma amostra de 3 microgramas de RNA total como material de partida. Os RNAs ribossômicos foram posteriormente removidos com precisão, utilizando o kit Ribo-Zero™ Gold (Human/Mouse/Rat, Illumina, EUA). Diferentes identificadores (índices) foram escolhidos para a construção das bibliotecas, conforme as instruções operacionais do kit NEBNext Ultra Directional RNA Library Prep para Illumina (NEB, Ipswich, EUA). De forma simplificada, o processo de construção da biblioteca foi dividido em quatro etapas principais. Primeiramente, após a remoção dos rRNAs, selecionaram-se os reagentes apropriados. Uma vez obtido o mRNA, aplicou-se o tampão de fragmentação para gerar os fragmentos desejados. Em seguida, esses fragmentos foram utilizados como moldes para a síntese da primeira fita de cDNA. A síntese da segunda fita de cDNA foi realizada com a adição de RNase H e dNTPs (dUTP). Na terceira etapa, após a eliminação inicial dos rRNAs, foi utilizado um kit de purificação para realizar a limpeza das amostras. A etapa seguinte envolveu uma sequência detalhada de procedimentos, incluindo a integração do Grupo Básico A e o reparo das extremidades. Por fim, o fragmento-alvo obtido por eletroforese em gel de agarose foi recuperado, e a amplificação por PCR (Qiagen, Alemanha) foi realizada para completar a construção da biblioteca.

Após a conclusão da construção da biblioteca, foi realizada a diluição quantitativa utilizando o fluorímetro Qubit 3.0 (Thermo Fisher Scientific, EUA), ajustando-se a concentração da biblioteca para 1 ng/μl. Com a fase de construção finalizada, o equipamento Agilent 2100 (Agilent, EUA) foi utilizado para medir o tamanho do inserto. A PCR quantitativa (qPCR) foi realizada com o equipamento CFX96 (Bio-Rad, EUA) e o kit fluorescente iQ SYBR GRN (Bio-Rad, EUA). A concentração efetiva da biblioteca foi quantificada com precisão (concentração efetiva > 10 nM) para assegurar a qualidade da biblioteca. Após a detecção e validação da biblioteca, diferentes bibliotecas foram agrupadas no *flow cell* de acordo com os requisitos de concentração efetiva e volume de dados-alvo. A geração de clusters no cBot foi seguida do sequenciamento em plataforma de alto rendimento Illumina (NovaSeq 6000, Illumina, EUA), com obtenção de leituras pareadas de 150 pb. Em seguida, foi realizada uma série de procedimentos, incluindo a aplicação do Grupo Básico A e a finalização do reparo das extremidades. A biblioteca foi então submetida à qPCR, desde que o tamanho do inserto estivesse dentro dos parâmetros esperados, sendo exigida uma concentração efetiva superior a 10 nM para garantir sua qualidade.

#### Sequenciamento em máquina

Após a finalização da construção das bibliotecas, as bibliotecas qualificadas foram agrupadas no *flow cell* de forma adequada, conforme a concentração efetiva estabelecida e os objetivos definidos para o sequenciamento. Em seguida, após a formação bem-sucedida dos clusters, as amostras foram submetidas à análise transcriptômica por sequenciamento de alto rendimento.

#### Análise transcriptômica por sequenciamento de alto rendimento

#### Pré-processamento dos dados e CQ:

As leituras brutas geradas pelo sequenciamento de alto rendimento foram fornecidas no formato FASTQ. Para obter leituras de alta qualidade adequadas às análises posteriores, foi realizado um processo de filtragem de qualidade. Inicialmente, utilizou-se o software fastp^17^ para CQ e remoção de adaptadores. Bases de baixa qualidade foram filtradas, resultando em um conjunto de leituras limpas de alta qualidade (*clean reads*). Em seguida, essas leituras limpas foram comparadas com sequências de rRNA utilizando o software SortMeRNA,^18^ sendo removidas aquelas que apresentaram correspondência. As leituras restantes foram mantidas para as análises subsequentes.

#### Alinhamento genômico e processamento dos transcritos:

O software Hisat2^19^ (http://www.psc.edu/user-resources/software/hisat2) foi utilizado para alinhar as *clean reads* ao genoma de referência, com os parâmetros padrão aplicados. A montagem dos transcritos foi realizada por meio do *software* StringTie, gerando novos isoformas de transcritos. Esses isoformas foram comparados com a anotação do genoma de referência utilizando o Cuffcompare, a fim de identificar transcritos conhecidos e novos.

#### Predição de lncRNAs:

A maioria dos lncRNAs não possui potencial codificador de proteínas. Foram selecionados transcritos com dois ou mais éxons e comprimento superior a 200 pb. Os lncRNAs candidatos foram identificados por meio da predição do potencial codificador utilizando as ferramentas CPC, CNCI, Pfam e PLEK.

#### Quantificação transcricional, análise de expressão diferencial, enriquecimento funcional e análise de agrupamento:

As *clean reads* com os rRNAs removidos foram alinhadas aos transcritos de referência (mRNAs e lncRNAs) utilizando o software Bowtie2,^20^ e os resultados foram armazenados em arquivos binários de alinhamento (formato BAM). O software *eXpress* foi utilizado para quantificar a abundância dos transcritos, fornecendo tanto a contagem de leituras quanto os valores de *fragments per kilobase of transcript per million* (FPKM).

Para experimentos com réplicas biológicas, a normalização dos dados foi realizada por meio da função estimateSizeFactors do pacote DESeq (2012) no R.^21^ A expressão diferencial foi avaliada com a função nbinomTest, que calcula os valores de p e as razões de mudança (*fold change*). Para experimentos sem réplicas biológicas, a análise de expressão diferencial foi conduzida com base no pacote edgeR (http://www.bioconductor.org/packages/release/bioc/html/edgeR.html),^22^ utilizando o teste de distribuição binomial negativa (NB). As contagens de leitura foram avaliadas quanto à significância estatística por meio desse teste, e os valores de CPM (*counts per million*) foram utilizados para estimar os níveis de expressão gênica.

Os genes diferencialmente expressos com valor de p inferior a 0,05 foram selecionados, e um teste de distribuição hipergeométrica foi realizado no software R para conduzir as análises de enriquecimento em ontologia gênica (*gene ontology*, GO) e na Kyoto Encyclopedia of Genes and Genomes (KEGG). Essas análises permitiram identificar as funções biológicas e vias mais impactadas pelos genes diferencialmente expressos (*differentially expressed genes*, DEGs).

#### Análise de splicing alternativo, polimorfismo de nucleotídeo único (SNP) e INDELs:

A análise de *splicing* alternativo foi realizada utilizando o *software* ASprofile.^23^ Os programas Samtools^24^ e BCFtools^25^ foram utilizados para identificar os sítios de SNPs e INDELs. Para detalhes sobre o procedimento específico, consulte o site do Samtools website (http://samtools.sourceforge.net/mpileup.shtml). Todos os *softwares* foram executados com os parâmetros padrão.

#### Identificação, quantificação e análise diferencial de circRNAs:

Com base no banco de dados circBase,^26^ os dados de sequenciamento foram inicialmente analisados utilizando o software CIRI^27^ para a predição de circRNAs. Os resultados preditos foram então comparados com o banco de dados para identificar tanto circRNAs conhecidos quanto novos. Os genes sobrepostos foram extraídos e anotados com base nas posições cromossômicas dos circRNAs.

A abundância de circRNAs foi quantificada utilizando RPM (leituras em junção por milhão de leituras). Para experimentos com replicação biológica, a normalização dos dados foi realizada por meio da função estimateSizeFactors do pacote DESeq (2012) no R, e os valores p e de mudança na expressão (*fold change*) foram calculados com a função nbinomTest. Para experimentos sem replicação, a expressão diferencial foi determinada utilizando o pacote edgeR (http://www.bioconductor.org/packages/release/bioc/html/edgeR.html), com base na distribuição NB.

CircRNAs com valores de p < 0,05 foram considerados diferencialmente expressos de forma significativa. As análises de enriquecimento funcional em GO e KEGG foram conduzidas com base nos genes sobrepostos, a fim de identificar as funções biológicas e vias mais afetadas pelos circRNAs diferencialmente expressos. Além disso, foi realizado o agrupamento hierárquico não supervisionado, e os padrões de expressão de circRNAs entre as amostras foram visualizados por meio de um mapa de calor (*heatmap*).

## Predição de interações entre circRNAs e miRNAs:

Como os circRNAs contêm múltiplos sítios de ligação para miRNAs, suas interações com miRNAs podem ser previstas utilizando ferramentas consolidadas de predição de genes-alvo de miRNAs. A função de um determinado circRNA pode, então, ser inferida com base na anotação funcional dos genes-alvo associados aos seus miRNAs correspondentes. Para espécies animais, as predições foram realizadas com o software miRanda.^28,29^ Para espécies vegetais, utilizou-se o software psRNATarget.^30^

### Tamanho amostral

Como este estudo se encontra em fase exploratória, adotou-se um tamanho amostral de n = 3, após cuidadosa consideração de custo-benefício e potencial de descoberta.

## Análise estatística

A expressão diferencial de um mesmo transcrito entre diferentes amostras foi calculada com base nos dados de RNA-seq. Para avaliar a variação na expressão, utilizou-se o valor de FoldChange como medida da alteração nos níveis de expressão de um transcrito entre dois grupos, enquanto o valor de p ou a *false discovery rate* (FDR — taxa de descoberta falsa, ou p-valor ajustado) foi utilizado para determinar a significância estatística. O valor de p de cada transcrito foi calculado e ajustado para múltiplas comparações. Transcritos foram considerados diferencialmente expressos de forma significativa quando o FoldChange foi superior a 2 e o valor de p foi inferior a 0,05.

Para comparações com replicações biológicas, os níveis diferenciais de expressão gênica foram calculados utilizando o teste de distribuição NB no pacote DESeq. A significância das diferenças nas contagens de leitura foi avaliada por meio do teste de distribuição NB. O valor de basemean foi utilizado para estimar os níveis de expressão gênica.^31^ Para comparações sem replicações biológicas, utilizou-se o pacote edgeR para análise e cálculo dos níveis de expressão gênica. A significância das diferenças nas contagens foi testada com base na distribuição NB, e os níveis de expressão gênica foram estimados por meio dos valores de CPM.

Para identificar variações entre diferentes amostras, a análise de expressão gênica diferencial é fundamental, uma vez que as principais diferenças se refletem no nível do transcrito. Portanto, a identificação de transcritos com expressão diferencial deve ser priorizada. Em seguida, devem ser realizadas análises abrangentes de enriquecimento funcional em KEGG e GO, a fim de investigar as vias biológicas e funções afetadas. A análise de enriquecimento em GO classifica os genes em três categorias: componente celular (CC), função molecular (MF) e processo biológico (BP), identificando funções biológicas significativamente associadas aos DEGs, por meio do teste exato de Fisher, considerando-se p < 0,05 como estatisticamente significativo. Da mesma forma, a análise de enriquecimento em KEGG utiliza dados genômicos para identificar vias enriquecidas e é realizada com o pacote clusterProfiler no RStudio (versão 4.3.1; R Foundation for Statistical Computing, Viena, Áustria), com um limiar de FDR < 0,05.

## Resultados

A Figura Central apresenta os dados mais relevantes deste estudo. Dois grupos experimentais foram analisados, cada um composto por três indivíduos (uma mulher e dois homens). A média de idade foi de 56 anos para o grupo caso e 54 anos para o grupo controle. Nenhuma comorbidade relevante foi identificada em ambos os grupos. O nível de expressão diferencial de cada transcrito foi avaliado utilizando o software DESeq, e a significância das diferenças nas contagens de leitura foi determinada por meio do teste de distribuição NB. Os níveis de expressão gênica foram estimados com base no valor de basemean.

### Análise dos transcritos com expressão diferencial de lncRNAs

**Figura 1:**
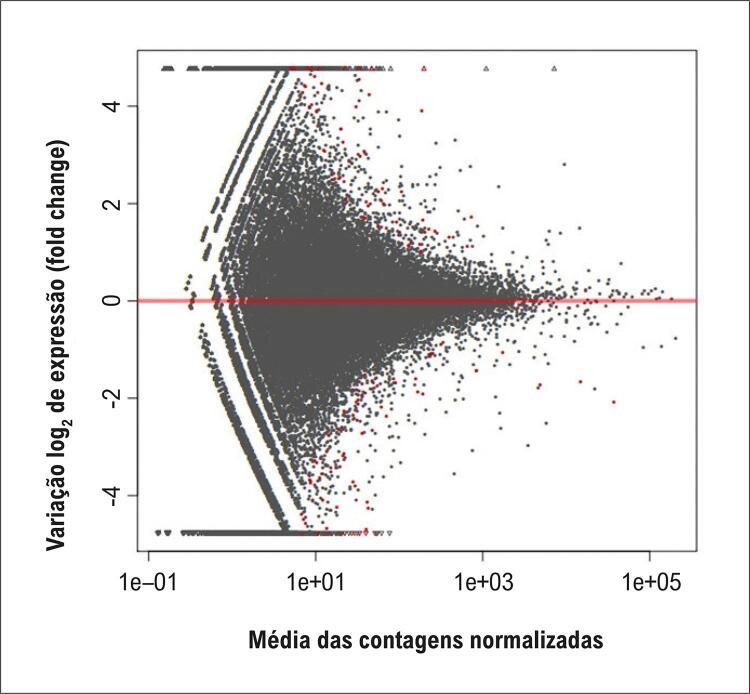
– Gráfico de média móvel dos transcritos com lncRNAs diferencialmente expressos.

**Figura 2:**
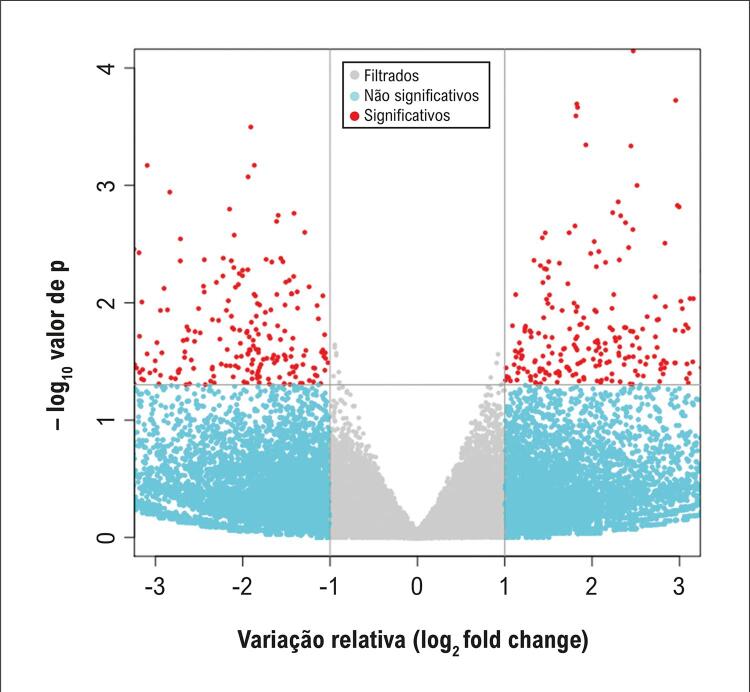
– Gráfico de vulcão dos transcritos com lncRNAs diferencialmente expressos. O gráfico exibe o log2 (razão de mudança) no eixo x e o –log10(p) no eixo y. Os pontos vermelhos indicam transcritos com expressão diferencial significativa, enquanto os pontos azuis e cinzas representam diferenças não significativas.

Dentre os 854 lncRNAs que apresentaram níveis de expressão alterados, 425 estavam regulados negativamente e 429, regulados positivamente. A Figura 1 e a Figura 2 apresentam, respectivamente, o gráfico de média móvel e o *volcano plot*, que visualizam os padrões de expressão diferencial dos transcritos de lncRNAs.

#### Mapa de calor da expressão diferencial de lncRNAs

**Figura 3:**
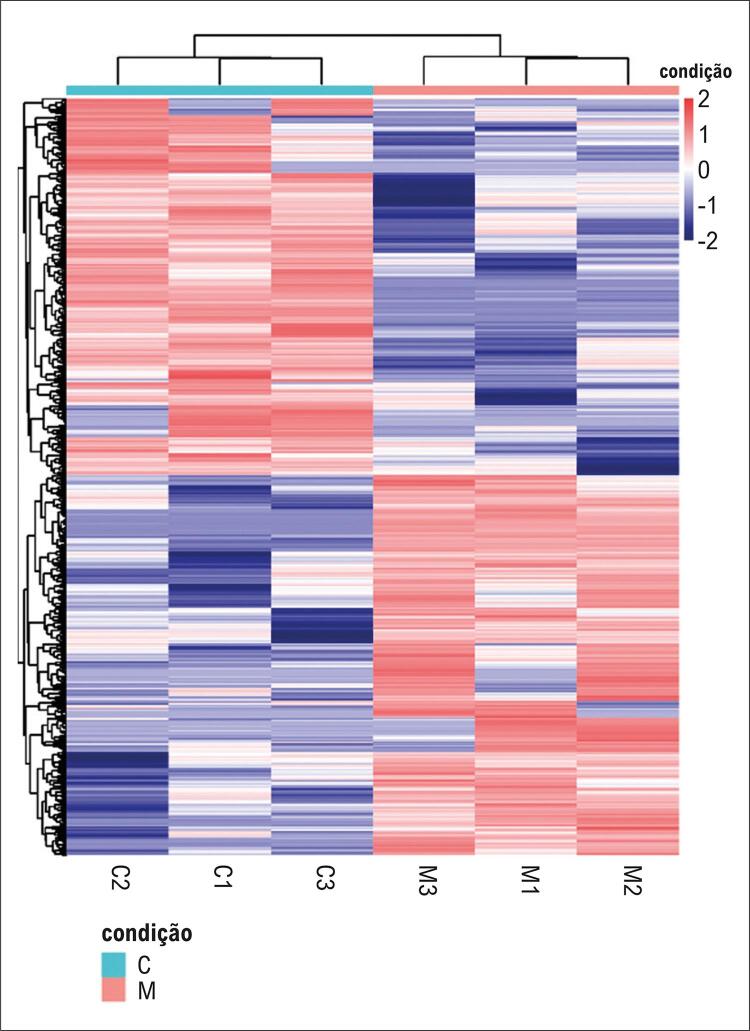
– Mapa de calor da expressão diferencial dos lncRNAs. (C: grupo controle; M: grupo com fluxo lento coronário).

O mapa de calor, gerado com o pacote *pheatmap* no R, demonstrou alta similaridade entre as amostras de um mesmo grupo, ao passo que foi observada expressão diferencial significativa entre os grupos. Os resultados são apresentados na Figura 3.

#### Análise de enriquecimento funcional dos genes-alvo

**Figura 4:**
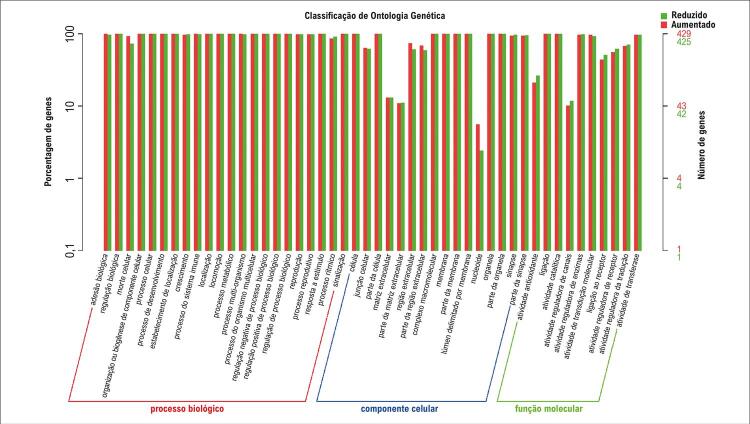
– Gráfico de barras de enriquecimento GO (48 principais termos). Os nomes dos termos GO estão representados no eixo x; o número de genes associados é indicado no eixo y à direita.

**Figura 5:**
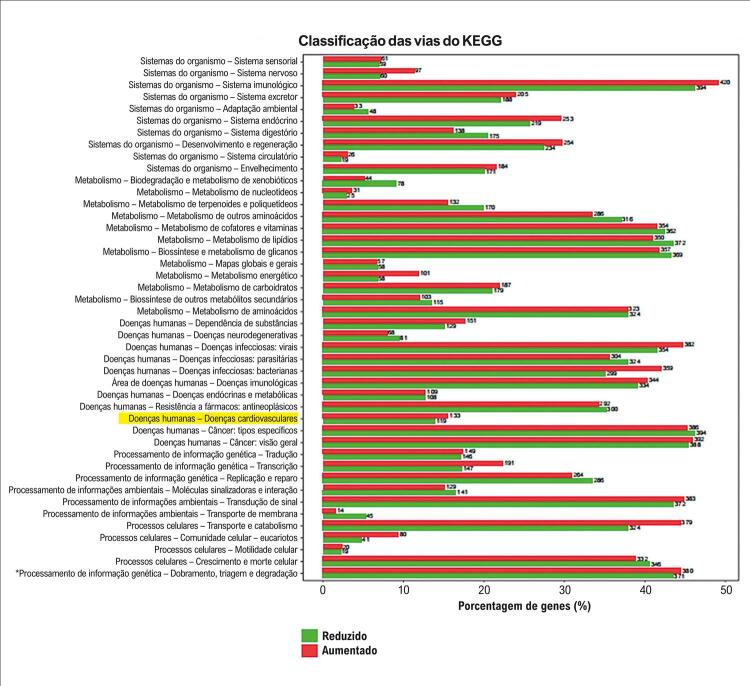
– Gráfico de enriquecimento KEGG. Os nomes das vias KEGG são exibidos no eixo y, e a porcentagem de genes associados é mostrada no eixo x.

Foi realizada uma análise de correlação entre lncRNAs e mRNAs com o objetivo de identificar transcritos de mRNAs associados aos lncRNAs. As correlações foram consideradas significativas quando o valor absoluto do coeficiente de correlação excedeu 0,8 e o valor de p foi inferior a 0,05. A anotação funcional, incluindo as análises de enriquecimento em GO e KEGG, foi conduzida com os mRNAs associados a cada lncRNA diferencialmente expresso. Os cálculos e os gráficos foram gerados para cada entrada gênica nos níveis 2 das categorias GO e KEGG, com base no status de regulação positiva ou negativa. Ver Figura 4 e Figura 5, nas quais R se refere ao ambiente gráfico utilizado.

A análise de enriquecimento funcional foi empregada para predizer os genes-alvo, identificando 48 entradas relevantes em GO. Os achados incluíram processos metabólicos e processos metabólicos celulares na categoria de BP; organelas intracelulares e citoplasma na categoria de CC; e atividade antioxidante, atividade reguladora de enzimas e atividade catalítica na categoria de MF. Para aprofundar a investigação sobre as possíveis funções desses lncRNAs, a análise de vias do KEGG revelou que sua expressão diferencial estava significativamente enriquecida em vias relacionadas a doenças cardiovasculares, distúrbios endócrinos, doenças metabólicas e doenças neurodegenerativas. Os 20 lncRNAs diferencialmente expressos mais relevantes estão apresentados na Tabela 1.

**Tabela 1:** – Top 20 lncRNAs diferencialmente expressos em pacientes com FLC

lncRNA	Regulação	*Fold change*	Valor de p
lnc-SLC46A2	Aumentado	307,6339927	8,16 × 10^-^^13^
lnc-DCAF4L1	Aumentado	301,9088015	2,37× 10^-^^23^
lnc-RAB23	Aumentado	293,4177516	1,72 × 10^-8^
lnc-LIPI	Aumentado	270,2219717	0,03287567
lnc-IGF2BP3	Aumentado	199,1040392	2,15 × 10^-7^
lnc-IFNGAS1	Aumentado	139,3191716	0,013423961
lnc-METTL2B	Aumentado	98,3100766	0,018769756
lnc-POLR3K	Aumentado	95,02233003	0,001041257
lnc-SOWAHB	Aumentado	84,70402792	0,000120964
lnc-PRKAB2	Aumentado	81,24009487	0,023770486
GAS5	Aumentado	81,15583069	0,000277837
LINC01949	Reduzido	0,019016197	5,39 × 10^-6^
lnc-CTSV	Reduzido	0,01855816	0,003755983
lnc-KCNE1B	Reduzido	0,016836927	0,013494903
lnc-WDR73	Reduzido	0,016808486	0,012120027
LINC00535	Reduzido	0,016156596	0,002651937
LINC00205	Reduzido	0,012744693	0,000840344
TUG1	Reduzido	0,012376483	0,014487006
lnc-SNRPN	Reduzido	0,009096652	0,003332174
lnc-PIGBOS1	Reduzido	0,008201513	0,001535038
lnc-SPI1	Reduzido	0,004864076	1,29 × 10^-6^

#### Resultados dos transcritos com expressão diferencial de mRNAs

**Figura 6:**
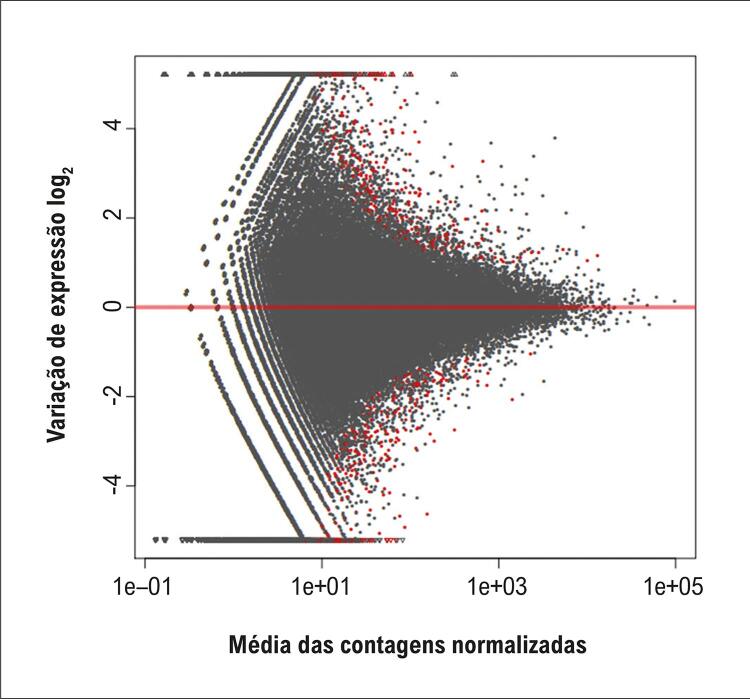
– Gráfico de média móvel dos transcritos com mRNAs diferencialmente expressos.

**Figura 7:**
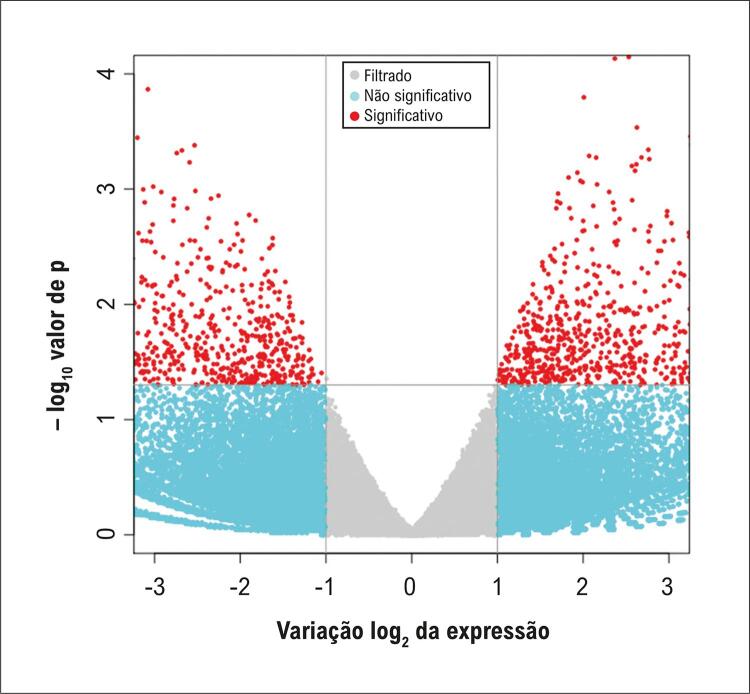
– Gráfico de vulcão dos transcritos com mRNAs diferencialmente expressos. O gráfico exibe o log2 da alteração de expressão (fold change) no eixo x e o –log10(p) no eixo y. Os pontos vermelhos e verdes indicam diferenças significativas; os pontos azuis e cinza representam diferenças não significativas.

Um total de 1.999 mRNAs apresentou expressão diferencial, dos quais 990 estavam regulados negativamente e 1.009 regulados positivamente. A Figura 6 e a Figura 7 exibem, respectivamente, o gráfico de média móvel e o *volcano plot*, os quais ilustram os perfis de expressão diferencial dos transcritos de mRNAs.

## Análise funcional dos transcritos com expressão diferencial

### Análise de enriquecimento em GO dos transcritos diferencialmente expressos:

A triagem das entradas de GO para transcritos com FoldChange superior a 2 resultou na identificação de 1.985 entradas significativamente enriquecidas entre os mRNAs diferencialmente expressos. A análise de agrupamento das 10 principais entradas, classificadas em ordem decrescente de –log_10_(p), envolveu predominantemente processos biológicos como defesa contra invasão viral, inibição da expressão do genoma viral, via de sinalização do interferon tipo I, disrupção da polarização mitocondrial e aumento da função de proteases.

Os mRNAs diferencialmente expressos também apresentaram enriquecimento significativo em 271 componentes celulares. As 10 principais entradas, com base na ordem decrescente de –log_10_(p), incluíram componentes como citoplasma, nucleoplasma, complexo quinase Atg1/ULK1, núcleo e locais de montagem de fagos, conforme identificado na análise de agrupamento.

Além disso, foram identificadas 531 funções moleculares com enriquecimento significativo. As 10 principais, classificadas por ordem decrescente de –log_10_(p), revelaram funções como ligação a proteínas, modulação da atividade de proteína quinase, atividade de transferase de ubiquitina-proteína e ligação a ácido ribonucleico.

**Figura 8:**
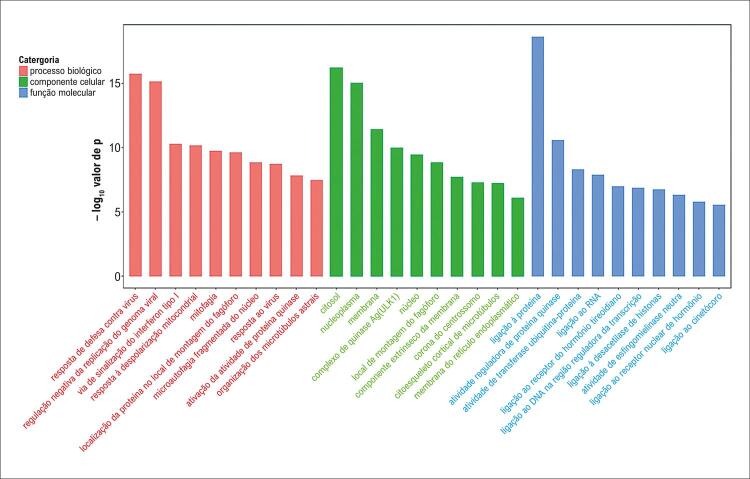
– Gráfico de barras do enriquecimento GO (30 principais termos). As barras vermelhas representam os 10 principais termos de processo biológico, as barras verdes os 10 principais termos de componente celular, e as barras azuis os 10 principais termos de função molecular. Os nomes dos termos GO estão no eixo x; o eixo y mostra os valores de –log10(p).

A Figura 8 apresenta o gráfico de barras com os 30 principais resultados de enriquecimento em GO. As entradas de GO com FoldChange transcricional superior a 2 foram filtradas separadamente para as três categorias, priorizando-se as 10 principais de cada uma com base nos respectivos valores de –log_10_(p) em ordem decrescente.

### Análise de enriquecimento em KEGG e mapeamento das vias dos DEGs:

**Figura 9:**
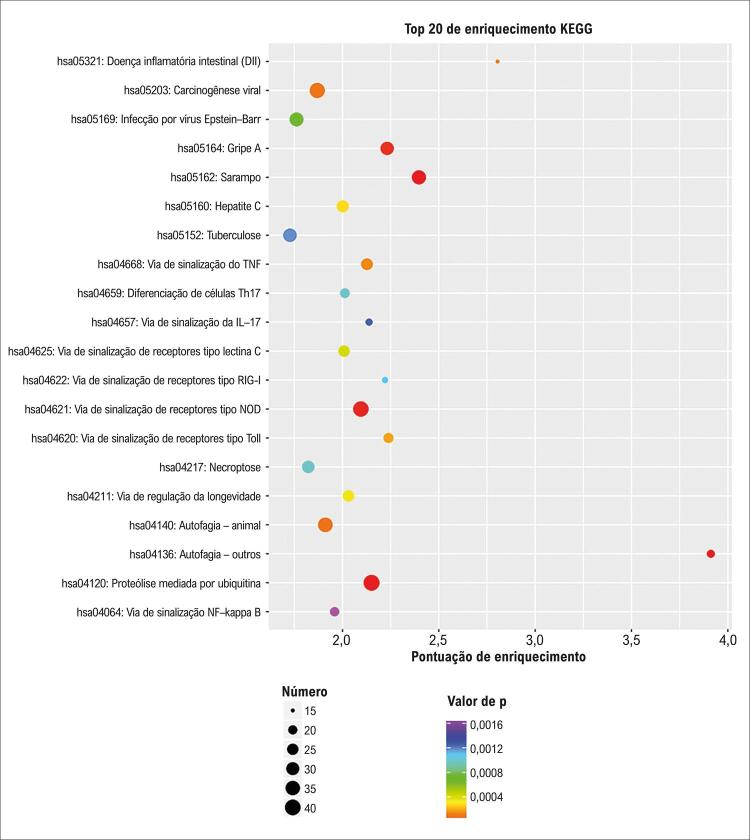
– Gráfico de bolhas do enriquecimento KEGG (20 principais termos). Os nomes das vias KEGG estão representados no eixo y; os valores de p são mostrados no eixo x. O tamanho das bolhas reflete o número de genes associados.

Após a triagem de 20 vias com FoldChange transcricional superior a 2, observou-se que os mRNAs diferencialmente expressos estavam significativamente enriquecidos em vias relacionadas à autofagia, sarampo, sistema ubiquitina–proteassoma, sinalização por receptores do tipo NOD, sinalização do fator de necrose tumoral (TNF), sinalização por receptores do tipo Toll (TLR) e via de sinalização NF-κB. A Figura 9 apresenta um *bubble plot* com os 20 principais resultados de enriquecimento em KEGG, com as vias classificadas em ordem decrescente de acordo com os respectivos valores de –log_10_(p).

### Top 20 mRNAs diferencialmente expressos:

A Tabela 2 apresenta os 20 mRNAs com maior expressão diferencial.

**Tabela 2:** – Top 20 mRNAs diferencialmente expressos em pacientes com FLC

Gene	Regulação	*Fold change*	Valor de p
SIRPB1	Aumentado	1424,292887	8,76 × 10^-5^
OTOF	Aumentado	749,2769265	0,033541446
CDC27	Aumentado	372,4377459	0,000308112
FCGR1B	Aumentado	293,4635502	0,002402462
HLA-DRB4	Aumentado	288,483249	0,000897273
MEF2C	Aumentado	279,7159676	7,28 × 10^-9^
ATG13	Aumentado	241,9462662	0,001107754
EPB41	Aumentado	173,0302956	0,004740905
ZNF45	Aumentado	140,5873565	0,000840983
SLC39A9	Aumentado	136,1707782	5,10 × 10^-5^
PBRM1	Reduzido	0,007401108	6,99 × 10^-5^
P2RX5	Reduzido	0,006490159	0,001694951
EPB41	Reduzido	0,006205606	0,033667873
SRRM1	Reduzido	0,006097372	1,07 × 10^-11^
ZNF445	Reduzido	0,006009509	0,005438717
CCR3	Reduzido	0,005964356	0,004797579
ALG13	Reduzido	0,004730269	6,94 × 10^-7^
ABCA7	Reduzido	0,004689293	0,019145994
KHSRP	Reduzido	0,004116791	0,00809256
MAP4K4	Reduzido	0,00314662	4,34 × 10^-6^

## Discussão

O sequenciamento transcriptômico de alto rendimento realizado neste estudo revelou um total de 854 lncRNAs diferencialmente expressos, dos quais 425 estavam regulados negativamente e 429 regulados positivamente. A análise de enriquecimento funcional identificou 48 entradas principais em GO. As predições de coexpressão e colocalização destacaram processos metabólicos e metabolismo celular na categoria de BP, bem como atividade antioxidante, regulação da atividade enzimática e atividade catalítica na categoria de MF. Na categoria de CC, componentes como junções celulares, organelas da matriz extracelular e organelas intracelulares foram associados ao citoplasma. A análise de vias do KEGG demonstrou que os lncRNAs diferencialmente expressos estavam significativamente enriquecidos em vias relacionadas a doenças cardiovasculares, distúrbios endócrinos, doenças metabólicas e doenças neurodegenerativas.

Os lncRNAs são reconhecidos por sua participação em processos fisiológicos e patológicos, exercendo funções regulatórias e estruturais em diversas atividades biológicas, como *imprinting* genético, controle epigenético, proliferação celular, processos de desenvolvimento, envelhecimento e apoptose, emergindo, assim, como moduladores-chave em diversas condições cardiovasculares.^12,32^ Além disso, os lncRNAs têm sido implicados na regulação do FLC e de suas complicações associadas.^12,32,33^

Em estudos relacionados ao DCAF4L1, gene codificador adjacente que é um componente chave do complexo de ubiquitina ligase CUL4-DDB1, foi demonstrado que esse gene influencia a progressão tumoral por meio da modulação de pontos de controle do ciclo celular e de vias de sinalização apoptótica. Um lncRNA possivelmente transcrito nesse lócus, referido como lnc-DCAF4L1 (ainda não oficialmente anotado), é hipoteticamente capaz de regular a inflamação imune. No entanto, seu mecanismo preciso requer validação experimental, por exemplo, por meio de técnicas como o *chromatin conformation capture* (4C-seq).^34^

É importante destacar que o lncRNA clássico GAS5 (*growth arrest-specific 5*) desempenha papéis distintos em diferentes redes patológicas. Ele regula fatores imunes, como a IL-10, por meio do mecanismo de RNA endógeno competidor (ceRNA) em doenças autoimunes, modula a via de sinalização da insulina em distúrbios metabólicos e contribui para processos neuroinflamatórios ao influenciar a ativação de micróglias em condições neurodegenerativas. Essa multifuncionalidade posiciona o GAS5 como um importante núcleo molecular para pesquisas interligadas entre diferentes doenças. Embora variações na expressão de genes codificadores de proteínas sejam reconhecidas, investigações adicionais são necessárias para elucidar suas implicações funcionais.^35,36^

Estudos demonstraram que lncRNAs como MALAT1 e NEAT1 desempenham um papel na promoção de respostas inflamatórias no FLC por meio de diferentes vias moleculares.^4,12,18^ O MALAT1 foi identificado como um biomarcador para a predição do fenômeno de não reperfusão durante a intervenção coronariana percutânea (pPCI) e como um potencial alvo terapêutico para o FLC.^37,38^ Adicionalmente, a correlação entre os níveis do lncRNA AF131217.1 e o FLC tem sido investigada, sugerindo um papel regulador desse lncRNA na inflamação induzida pelo FLC por meio da modulação do fator semelhante ao Krüppel 4 (KLF4).^38^ No entanto, no presente estudo, MALAT1 e NEAT1 não foram identificados como tendo funções regulatórias em pacientes com FLC.

Outros lncRNAs diferencialmente expressos identificados neste estudo — incluindo lnc-SLC46A2, lnc-RAB23 e lnc-LIPI (Tabela 1), com exceção de lnc-DCAF4L1 e GAS5 — ainda não foram previamente descritos na literatura. A relação entre esses lncRNAs e o FLC permanece indefinida e requer investigações adicionais para ser validada. Mais estudos são necessários para esclarecer os mecanismos específicos pelos quais os lncRNAs contribuem para o FLC e para explorar seu potencial como alvos terapêuticos.

Dentre os 1.999 mRNAs diferencialmente expressos, 990 estavam regulados negativamente e 1.009 regulados positivamente. De acordo com a análise de agrupamento em GO das 10 principais entradas, classificadas por –log_10_(p), os principais processos biológicos incluíram resposta à infecção viral, regulação negativa da expressão do genoma viral, via de sinalização do interferon tipo I, despolarização mitocondrial e ativação da atividade de proteases. Os principais componentes celulares envolvidos foram o citoplasma, nucleoplasma, complexo quinase Atg1/ULK1, núcleo e local de montagem de fagos. As funções moleculares estavam predominantemente relacionadas à ligação a proteínas, regulação da atividade de proteína quinase, atividade de transferase de ubiquitina-proteína e ligação a RNA. A análise de KEGG mostrou que os mRNAs diferencialmente expressos estavam significativamente enriquecidos nas vias de sinalização dos TLR e na via de sinalização NF-κB.

O estudo conduzido por Coto et al.^37^ identificou que a variação em NFKBIZ é um fator de risco independente para DAC de início precoce. A pesquisa incluiu 609 homens com DAC de início precoce e 423 homens saudáveis no grupo controle. Nenhuma diferença significativa entre os grupos foi observada nas frequências alélicas ou genotípicas de NFKB1 rs28362491 (−94 delATTG) ou NFKBIA rs8904; no entanto, a frequência da deleção NFKBIZ rs3217713 foi significativamente maior no grupo com DAC em comparação ao grupo controle.^37^ Em nosso estudo, a análise KEGG indicou que a via de sinalização NF-κB desempenha um papel na variação da expressão de mRNAs, sugerindo que a inflamação pode ser um dos mecanismos patológicos subjacentes ao FLC. No entanto, as pesquisas sobre a relação entre NFKBIZ e FLC ainda estão em estágio inicial. Os mRNAs regulados positivamente e negativamente identificados neste estudo (Tabela 2) ainda não foram previamente associados ao FLC na literatura existente. São necessários mais estudos para elucidar os mecanismos específicos que vinculam esses mRNAs ao FLC.

Diferentemente de pesquisas anteriores, que frequentemente se concentraram em lncRNAs ou miRNAs isoladamente, este estudo oferece uma perspectiva mais abrangente ao integrar os perfis de expressão de lncRNAs e mRNAs, bem como suas interações no contexto do FLC. Embora estudos anteriores já tenham sugerido a participação de lncRNAs como MALAT1 em respostas inflamatórias e na disfunção endotelial, nossos achados destacam especificamente a desregulação de lncRNAs como ANRIL, MALAT1 e LINC00305 em pacientes com FLC, oferecendo novas perspectivas sobre potenciais alvos terapêuticos e biomarcadores.

### Limitações do estudo

A principal limitação deste estudo é o pequeno tamanho amostral, com apenas três indivíduos em cada grupo. Esse número limitado pode não capturar adequadamente a variabilidade biológica, reduzindo o poder estatístico e, consequentemente, comprometendo a generalização dos achados. Além disso, o estudo não incluiu outros tipos de RNAs não codificantes, como os microRNAs, que também podem ser relevantes. Essas limitações indicam que estudos futuros com coortes maiores e mais diversas são essenciais para validar os resultados obtidos e para elucidar de forma mais completa os mecanismos subjacentes ao FLC.

## Conclusão

Em conclusão, este estudo confirma que os lncRNAs — definidos como transcritos com mais de 200 pares de bases — desempenham papéis cruciais no FLC, com mecanismos potenciais envolvendo, principalmente, a modulação de vias inflamatórias e a regulação da função endotelial. No entanto, os lncRNAs e mRNAs regulados positiva e negativamente identificados neste estudo ainda não foram previamente associados ao FLC na literatura. São necessárias pesquisas adicionais para esclarecer os mecanismos específicos que sustentam a relação entre esses transcritos e o FLC.
