# Supplementary material for: Análise Transcriptômica por Sequenciamento de Alto Rendimento da Expressão de lncRNAs e mRNAs em Pacientes com Fluxo Lento Coronário
Source: Arq Bras Cardiol. 2025 Aug 22;122(9):e20240847. [Article in Portuguese] doi: 10.36660/abc.20240847 (PMC12677524; doi:10.36660/abc.20240847)
Supplement: Translation

# Translation: Transcriptome High-Throughput Sequencing Analysis of lncRNA and mRNA Expression in Patients with Coronary Slow Flow

**DOI:** 10.36660/abc.20240847i

**Authors:** Haibing Jiang, Yi Yang, Xueqin Zhai, Lijing Zhang, Aerziya Kahaerjiang

**Affiliations:** 1 China Hospital of Traditional Chinese Medicine Affiliated to Xinjiang Medical University, Urumqi – China; 2 China Xin Jiang Medical University, Urumqi, Xinjiang – China

**Keywords:** High-Throughput Nucleotide Sequencing, Messenger RNA, Long Noncoding RNA, Inflamação

## Abstract

**Background:**

Long non-coding RNAs (lncRNAs) and microRNAs (miRNAs) are believed to play key roles in the pathophysiology of coronary slow flow (CSF).

**Objectives:**

This study aimed to explore the complex biological networks involved in CSF using whole-transcriptome sequencing, with the goal of identifying potential diagnostic biomarkers and therapeutic targets.

**Methods:**

Whole-transcriptome sequencing was performed on samples from three patients with CSF and three matched control subjects. A p-value < 0.05 was considered statistically significant.

**Results:**

A total of 854 lncRNAs were differentially expressed, with 425 downregulated and 429 upregulated. KEGG pathway analysis showed significant enrichment of lncRNAs in pathways associated with cardiovascular diseases, endocrine and metabolic disorders, and neurodegenerative disease progression. Additionally, 1,999 mRNAs were differentially expressed, including 990 downregulated and 1,009 upregulated. Molecular function analysis identified roles in protein binding, regulation of kinase activity, ubiquitin-protein transferase activity, and RNA binding. KEGG analysis indicated that the differentially expressed mRNAs were primarily involved in autophagy, measles, ubiquitin-mediated proteolysis, the NOD-like receptor signaling pathway, the tumor necrosis factor (TNF) signaling pathway, the toll-like receptor (TLR) signaling pathway, and the NF-κB signaling pathway.

**Conclusions:**

Differentially expressed mRNAs were significantly enriched in KEGG pathways related to autophagy, measles, and ubiquitin-mediated degradation, as well as in signaling cascades involving NOD-like receptors, TNF, TLR, and NF-κB. Further studies are required to validate these findings.

## Introduction

Coronary slow flow (CSF) is thought to be closely associated with the onset of various manifestations of the CSF phenomenon.^1-5^ This condition has been linked to several contributing factors, including psychiatric disorders, serum salusin-β levels, homocysteine, cystatin C, body mass index, and systemic immune-inflammation.^2,3,6,7^ The inflammatory response may play a central role in multiple pathophysiological processes, such as microvascular obstruction and microthrombus formation, which are considered key elements in the pathogenesis of coronary microcirculatory dysfunction.^8-11^

Recent studies have shown that certain long non-coding RNAs (lncRNAs) can regulate downstream inflammatory factors, thereby influencing the development of CSF.^9,12^ Danaii et al. proposed that microRNAs (miRNAs) may serve as potential biomarkers for diagnosing CSF and monitoring the progression of coronary artery disease (CAD) in affected patients.^13^ Based on these findings, lncRNAs and miRNAs appear to play significant roles in the pathophysiology of CSF. However, given the complexity of the condition and the diverse functions of non-coding RNAs in the cardiovascular system, focusing on only a few molecules may not provide a comprehensive understanding of the underlying mechanisms.

Whole-transcriptome analysis offers a broader perspective by enabling the identification of key lncRNAs and miRNAs involved in CSF and their potential interactions. This approach may contribute to a deeper understanding of the molecular basis of CSF and support future clinical applications.

RNA sequencing (RNA-seq) has been widely used to investigate gene expression profiles in cardiac tissues and cells, providing insights into molecular changes associated with conditions such as CSF and Takotsubo syndrome, a form of coronary microvascular disease.^14,15^ A transcriptomic study of CSF using RNA extracted from peripheral blood monocytes revealed differential gene expression patterns and an association with inflammation.^16^ However, the pathogenesis of CSF remains complex and poorly understood, and only a few studies to date have applied transcriptome sequencing specifically to CSF.

Therefore, the present study aimed to investigate the transcriptomic landscape of patients with CSF through high-throughput sequencing of peripheral blood leukocytes in order to identify genes and pathways potentially involved in the development of CSF.

## Methods

### Subjects

This study, supported by grant number 2016XE0113, was conducted in accordance with the ethical principles outlined in the Declaration of Helsinki. All participants were fully informed about the study objectives and provided written informed consent. The study protocol was approved by the Research Ethics Committee, ensuring the protection of participants’ rights and welfare.

Three patients diagnosed with CSF based on coronary angiography, along with three individuals with normal coronary angiography findings, were enrolled at the Traditional Chinese Medical Hospital of Xinjiang Uygur Autonomous Region. These individuals were assigned to the case group and control group, respectively. All participants were initially suspected of having uncomplicated coronary heart disease and had no history of diabetes, cardiomyopathy, vasculitis, connective tissue disorders, or endocrine diseases.

### Diagnostic criteria

CSF was defined as a delayed perfusion of any major distal coronary vessel exceeding three cardiac cycles, as observed during coronary angiography.

### Exclusion criteria

Participants were selected according to strict inclusion and exclusion criteria to ensure the reliability and validity of the study findings. Patients were excluded if they presented with hypotension (systolic blood pressure ≤ 90 mmHg at the time of angiography) in the absence of vasodilator medication use, or if they had a heart rate < 60 beats per minute without the influence of heart rate–modifying drugs. Individuals who had experienced an acute myocardial infarction or acute coronary syndrome — including unstable angina pectoris — within the previous month were also excluded. In addition, patients with any coronary artery stenosis > 50% were not eligible for inclusion. Clinical data, laboratory indices, and blood samples were collected and analyzed from all participants.

### Experimental methods

#### Database construction and sequencing process

Total RNA was extracted from blood samples following centrifugation, lysis, and precipitation. The integrity of the isolated RNA was evaluated using standard analytical procedures, followed by a quality assessment. Ribosomal RNAs (rRNAs) were subsequently removed to enrich for mRNAs and non-coding RNAs. The remaining RNA was fragmented and reverse-transcribed into complementary DNA (cDNA). Next, blunt-ended cDNAs underwent adaptor ligation and tailing. The resulting cDNA library was size-selected using agarose gel electrophoresis, and the band corresponding to the target size was excised. The selected cDNA fragments were then amplified by polymerase chain reaction (PCR) and subjected to high-throughput sequencing using an automated sequencing platform.

#### Sample collection and processing of whole blood specimens

The initial step involved accurate documentation and identification of 4 mL whole blood samples collected from diagnosed patients, with each sample labeled according to the patient registry. Following centrifugation, plasma and cellular components were separated. The plasma, located in the upper layer after centrifugation, was transferred into 1.5 mL Eppendorf tubes, with each tube receiving 400 μL of plasma. The tubes were then sealed and stored in an ultra-low temperature freezer at –80°C for preservation.

Centrifugation was performed at 3,000 rpm for 10 minutes to ensure effective separation of plasma. The resulting cellular pellet was resuspended in ammonium-chloride-potassium (ACK) Lysis Buffer (Bioseth, Zhenjiang, China) and transferred into fresh 1.5 mL Eppendorf tubes. Each patient’s pellet was evenly divided into two groups for further analysis, ensuring sample integrity for subsequent procedures.

The Eppendorf tubes containing the resuspended cells were incubated at room temperature for 5 minutes to promote lysis. Centrifugation was then carried out to separate the components. To improve lysis efficiency, treatment with ACK Lysis Buffer was repeated two to three times, ensuring thorough cell disruption. After the final lysis step, the samples were rinsed with physiological saline to remove residual buffer.

For RNA extraction, leukocytes in one set of 1.5 mL Eppendorf tubes were processed using a TRIzol-based resuspension–precipitation protocol with TRIzol reagent (Invitrogen, USA). Another set of leukocyte-containing tubes was left untreated and stored at –80°C for future use.

#### RNA sample testing

The integrity and purity of the RNA samples were evaluated through a multi-step quality control (QC) process. Initially, agarose gel electrophoresis was used to detect degradation and contamination. This method separates nucleic acid fragments by size, allowing for the visual assessment of RNA integrity. Next, spectrophotometric analysis was performed to determine RNA purity, with the A260/A280 ratio used as a standard indicator. High-precision quantification of RNA concentration was then conducted using the Qubit® 3.0 Fluorometer, ensuring accurate measurement of RNA yield. Finally, the Agilent 2100 Bioanalyzer with the RNA Nano 6000 Assay Kit was employed to assess RNA integrity. This analysis generated an RNA integrity number (RIN), which provides a quantitative measure of RNA quality.

#### Construction and testing of the library

For the lncRNA library construction, a 3-microgram sample of total RNA was chosen as the starting material. The ribosomal RNAs were subsequently removed from the samples with precision, facilitated by the Ribo-Zero™ Gold Kit (Human/Mouse/Rat, Illumina, USA). Different index labels were selected respectively for library construction based on the operating instructions of the NEBNext Ultra Directional RNA Library Prep Kit for Illumina (NEB, Ipswich, USA). In simpler terms, the whole process of the library construction consisted of four parts. Firstly, starting with the essential step of rRNA removal, one should then proceed to select the pertinent reagent kits. After securing the mRNA, the application of a fragmentation buffer yields the desired mRNA fragments. Secondly, fragments were used as templates, then the first-strand cDNA was synthesized. The second-strand cDNA synthesis was completed by adding RNase H and dNTPs (dUTP). Thirdly, the rRNAs were initially eliminated, after which a purification kit was employed to carry out the purification. This was followed by a cascade of detailed operations, including the integration of Basic Group A and the subsequent end repair. Finally, the target fragment in the agarose gel electrophoresis was recovered, and PCR amplification (Qiagen, Germany) was performed to complete the construction of the library.

Upon the culmination of library construction, quantitative dilution utilizing the Qubit 3.0 Fluorometer (Thermo Fisher Scientific, USA) was conducted, resulting in a library concentration adjusted to 1 ng/μL. With the library construction phase concluded, the Agilent 2100 (Agilent, USA) was engaged to measure the insert size. Quantitative PCR (qPCR) was performed using the Bio-RAD CFX96 (Bio-Rad, USA) fluorescent quantitative PCR instrument and the Bio-RAD iQ SYBR GRN Kit (Bio-Rad, USA). The effective concentration of the library was accurately quantified (effective concentration > 10 nM) to ensure the quality of the library. After qualified library detection, different libraries were pooled to the flow cell according to the requirements of effective concentration and target data volume. cBot clusters were sequenced using the Illumina high-throughput sequencing platform (NovaSeq 6000, Illumina, USA), and 150 bp paired-end reads were obtained. Subsequently, a chain of operations was implemented, such as the application of Basic Group A and the finalization of end repair. The library was then positioned for qPCR, provided the insert size aligned with expectations, with the library’s effective concentration required to surpass 10 nM to ensure its quality.

#### Machine sequencing

Once the library construction reached its final stage, the qualified libraries were adeptly pooled onto the flow cell, conforming to the designated effective concentration and the set sequencing goals. Thereafter, the samples underwent comprehensive transcriptome high-throughput sequencing analysis once the clusters were successfully established.

#### Transcriptome high-throughput sequencing analysis

#### Data preprocessing and QC:

The raw reads generated from high-throughput sequencing were provided in FASTQ format. To obtain high-quality reads suitable for downstream analysis, quality filtering was performed. First, the fastp software^17^ was used for QC and adapter removal. Low-quality bases were filtered out, resulting in a set of high-quality clean reads. The clean reads were then compared against rRNA sequences using SortMeRNA software,^18^ and any matched reads were removed. The remaining reads were retained for subsequent analyses.

#### Genome alignment and transcript splicing:

Hisat2^19^ (http://www.psc.edu/user-resources/software/hisat2) was used to align the clean reads to the reference genome, with default parameters applied. Transcript assembly was performed using StringTie software, generating new transcript isoforms. These were compared against the reference genome annotation using Cuffcompare to identify known and novel transcripts.

#### Prediction of lncRNAs:

Most lncRNAs lack protein-coding potential. Transcripts with two or more exons and a length greater than 200 bp were selected. Candidate lncRNAs were identified through coding potential prediction using CPC, CNCI, Pfam, and PLEK.

#### Transcriptional quantification, differential expression analysis, functional enrichment, and cluster analysis:

Clean reads with rRNA removed were aligned to reference transcripts (mRNA and lncRNA) using Bowtie2,^20^ and the results were stored in binary alignment (BAM) files. eXpress was used to quantify transcript abundance, providing both read counts and fragments per kilobase of transcript per million (FPKM) values.

For experiments with biological replicates, data normalization was performed using the estimateSizeFactors function in the DESeq (2012) R package.^21^ Differential expression was assessed using the nbinomTest function to calculate *p*-values and fold changes. For experiments without biological replicates, differential expression analysis was conducted based on the edgeR package (http://www.bioconductor.org/packages/release/bioc/html/edgeR.html),^22^ using the negative binomial distribution test. Read counts were evaluated for statistical significance using a negative binomial (NB) test, and counts per million (CPM) values were used to estimate gene expression levels.

Differential genes with a p-value less than 0.05 were selected, and a hypergeometric distribution test was performed in R to conduct gene ontology (GO) and Kyoto Encyclopedia of Genes and Genomes (KEGG) enrichment analyses. These analyses were used to identify the biological functions and pathways most affected by the differentially expressed genes (DEGs). Additionally, the pheatmap function in R was employed to perform unsupervised hierarchical clustering of the DEGs, and a heatmap was generated to visualize their expression patterns across different samples.

#### Alternative splicing, single nucleotide polymorphism (SNP), and INDEL analysis:

Alternative splicing analysis was conducted using ASprofile software.^23^ Samtools^24^ and BCFtools^25^ were used to identify SNP and INDEL sites. For details on the specific procedure, please refer to the Samtools website (http://samtools.sourceforge.net/mpileup.shtml). All software was run using default parameters.

#### circRNA identification, quantification, and differential analysis:

Based on the circBase database,^26^ sequencing data were first analyzed using CIRI software^27^ to predict circRNAs. The predicted results were then compared with the database to identify both known and novel circRNAs. Overlapping genes were extracted and annotated based on the chromosomal positions of circRNAs.

CircRNA abundance was quantified using RPM (spliced reads per million reads). For experiments with biological replication, data normalization was performed using the estimateSizeFactors function in the DESeq (2012) R package, and p-values and fold change values were calculated using the nbinomTest function. For experiments without replication, differential expression was determined using the edgeR package (http://www.bioconductor.org/packages/release/bioc/html/edgeR.html), based on the negative binomial distribution.

CircRNAs with p-values < 0.05 were considered significantly differentially expressed. GO and KEGG enrichment analyses were conducted based on overlapping genes to identify the biological functions and pathways most affected by the differentially expressed circRNAs. In addition, unsupervised hierarchical clustering was performed, and circRNA expression patterns across samples were visualized using a heat map.

## Prediction of circRNA–miRNA target interactions:

Since circRNAs contain multiple miRNA binding sites, their interactions with miRNAs can be predicted using established miRNA target gene prediction tools. The function of a given circRNA can then be inferred based on the functional annotation of its associated miRNA target genes. For animal species, predictions were performed using miRanda software.^28,29^ For plant species, predictions were carried out using psRNATarget software.^30^

### Sample size

As this study was in the exploratory phase, a sample size of n = 3 was adopted after careful consideration of cost-effectiveness and discovery potential.

## Statistical analysis

Differential expression of the same transcript among different samples was calculated using RNA-seq data. To assess expression variation, FoldChange was used to measure the change in expression levels of a given transcript between two samples, while the p-value or false discovery rate (FDR) (adjusted p-value) was used to evaluate statistical significance. Each transcript’s p-value was calculated and adjusted for multiple testing. Transcripts were considered significantly differentially expressed if the FoldChange was greater than 2 and the p-value was less than 0.05.

For comparisons with biological replication, gene differential expression levels were calculated using the negative binomial distribution test in the DESeq software package. The significance of read count differences was assessed using the NB distribution test. The basemean value was used to estimate gene expression levels.^31^ For comparisons without biological replication, the edgeR software package was used to analyze and calculate gene expression levels. The significance of read differences was tested using the NB distribution, and gene expression levels were estimated using the CPM value.

To discern variations among different samples, gene differential expression analysis is pivotal, as differences are primarily reflected at the transcript level. Therefore, identifying transcripts with differential gene expression should be prioritized. Subsequently, comprehensive KEGG pathway enrichment and GO functional enrichment analyses should be performed to investigate the biological pathways and functions affected. GO enrichment analysis categorizes genes into cellular component (CC), molecular function (MF), and biological process (BP), identifying significant biological functions associated with DEGs using Fisher’s exact test, with p < 0.05 indicating statistical significance. Similarly, KEGG enrichment analysis uses genomic data to identify enriched pathways, and is performed using the clusterProfiler package in RStudio (version 4.3.1; R Foundation for Statistical Computing, Vienna, Austria), with an FDR < 0.05 threshold.

## Results

Central Illustration presents the most important data from this study. There were two experimental groups, each consisting of three individuals (one female and two males). The mean age was 56 years for the case group and 54 years for the control group. No notable comorbidities were identified in either group. The level of differential gene expression for each transcript was assessed using the DESeq software, and the significance of read count differences was evaluated using the NB test. Gene expression levels were estimated based on the basemean value.

### Analysis of transcripts with differential expression of lncRNA

Among the 854 lncRNAs that displayed altered expression levels, 425 were downregulated, while 429 were upregulated. Figure 1 and Figure 2 present the moving average chart and the volcano plot, respectively, which visualize the differential expression patterns of the lncRNA transcripts.

**Figure 1:** – Moving average chart of transcripts with differentially expressed lncRNAs.

**Figure 2:** – Volcano plot of transcripts with differentially expressed lncRNAs. The plot displays log2(fold change) on the x-axis and –log10(p-value) on the y-axis. Red dots indicate significantly differentially expressed transcripts, while blue and grey dots represent non-significant differences.

#### Heat map of differential expression of lncRNA

The heat map, generated using the pheatmap package in R, demonstrated high similarity between samples within the same group, while significant inter-group differential expression was observed. Results are shown in Figure 3.

**Figure 3:** – Heat map of differentially expressed lncRNAs (C: control group; M: coronary slow flow group).

#### Functional enrichment analysis of target genes

A correlation analysis between lncRNAs and mRNAs was conducted to identify lncRNA-associated mRNA transcripts. Correlations were considered significant if the absolute value of the correlation coefficient exceeded 0.8 and the p-value was less than 0.05. Functional annotation, including GO and KEGG enrichment analyses, was performed on the mRNAs associated with each differentially expressed lncRNA. Calculations and plots were generated for each gene entry at the GO and KEGG level 2 categories, based on upregulation and downregulation status. See Figure 4 and Figure 5, where R refers to the plotting environment.

**Figure 4:** – GO enrichment bar plot (top 48 terms). GO term names are shown on the x-axis; the number of associated genes is indicated on the right y-axis.

**Figure 5:** – KEGG enrichment plot. KEGG pathway names are displayed on the y-axis, and the percentage of associated genes is shown on the x-axis.

Functional enrichment analysis was used to predict the target genes, identifying 48 key GO entries. The findings included metabolic processes and cellular metabolic processes in the BP category; intracellular organelles and cytoplasm within the CC category; and antioxidant activity, enzyme regulatory activity, and catalytic activity in the MF category. To further explore the potential functions of these lncRNAs, KEGG pathway analysis revealed that their differential expression was significantly enriched in pathways related to cardiovascular diseases, endocrine disorders, metabolic diseases, and neurodegenerative diseases. Top 20 differentially expressed lncRNAs are presented in Table 1.

**Table 1:** – Top 20 differentially expressed lncRNAs in patients with CSF

lncRNA	Regulation	Fold change	p-value
lnc-SLC46A2	Up	307.6339927	8.16 × 10^-13^
lnc-DCAF4L1	Up	301.9088015	2.37× 10^-23^
lnc-RAB23	Up	293.4177516	1.72 × 10^-8^
lnc-LIPI	Up	270.2219717	0.03287567
lnc-IGF2BP3	Up	199.1040392	2.15 × 10^-7^
lnc-IFNGAS1	Up	139.3191716	0.013423961
lnc-METTL2B	Up	98.3100766	0.018769756
lnc-POLR3K	Up	95.02233003	0.001041257
lnc-SOWAHB	Up	84.70402792	0.000120964
lnc-PRKAB2	Up	81.24009487	0.023770486
GAS5	Up	81.15583069	0.000277837
LINC01949	Down	0.019016197	5.39 × 10^-6^
lnc-CTSV	Down	0.01855816	0.003755983
lnc-KCNE1B	Down	0.016836927	0.013494903
lnc-WDR73	Down	0.016808486	0.012120027
LINC00535	Down	0.016156596	0.002651937
LINC00205	Down	0.012744693	0.000840344
TUG1	Down	0.012376483	0.014487006
lnc-SNRPN	Down	0.009096652	0.003332174
lnc-PIGBOS1	Down	0.008201513	0.001535038
lnc-SPI1	Down	0.004864076	1.29 × 10^-6^

#### Results of transcripts with differential mRNA expression

A total of 1,999 mRNAs were found to be differentially expressed, including 990 that were downregulated and 1,009 that were upregulated. Figure 6 and Figure 7 display the moving average chart and the volcano plot, respectively, which illustrate the differential expression profiles of the mRNA transcripts.

**Figure 6:** – Moving average chart of transcripts with differentially expressed mRNAs.

**Figure 7:** – Volcano plot of transcripts with differentially expressed mRNAs. The plot displays log2(fold change) on the x-axis and –log10(p-value) on the y-axis. Red and green dots indicate significant differences; blue and gray dots represent non-significant differences.

## Functional analysis on transcripts with differential expression

### GO enrichment analysis of differentially expressed transcripts:

The screening of GO entries for transcripts with a FoldChange greater than 2 resulted in the identification of 1,985 entries significantly enriched among the differentially expressed mRNAs. Clustering analysis of the top 10 entries, ranked by decreasing –log_10_(p-values), primarily involved biological processes such as defense against viral invasion, inhibition of viral genome expression, type I interferon signaling pathway, disruption of mitochondrial polarization, and enhancement of protease function.

The differentially expressed mRNAs were also significantly enriched in 271 cellular components. The top 10 entries, based on decreasing –log_10_(p-values), included components such as the cytoplasm, nucleoplasm, Atg1/ULK1 kinase complex, nucleus, and phage assembly sites, as identified through clustering analysis.

Additionally, 531 molecular functions were found to be significantly enriched. The top 10 entries, ranked by decreasing –log_10_(p-values), revealed molecular functions such as protein binding, modulation of protein kinase activity, ubiquitin-protein transferase activity, and ribonucleic acid binding.

Refer to Figure 8 for the bar plot illustrating the top 30 GO enrichment results. GO entries with transcriptional FoldChange greater than 2 were filtered separately for the three categories, with the top 10 entries from each category prioritized based on their –log_10_(p-values) in descending order.

**Figure 8:** – GO enrichment bar plot (top 30 terms). Red bars represent the top 10 biological process terms, green bars the top 10 cellular component terms, and blue bars the top 10 molecular function terms. GO term names are on the x-axis; –log10(p-value) is on the y-axis.

### KEGG enrichment analysis and pathway network plotting of DEGs:

Following the screening of 20 pathway entries with a transcriptional FoldChange greater than 2, differentially expressed mRNAs were found to be significantly enriched in pathways related to autophagy, measles, the ubiquitin–proteasome system, NOD-like receptor signaling, tumor necrosis factor (TNF) signaling, toll-like (TLR) signaling, and the NF-κB signaling pathway. Figure 9 presents a bubble plot of the top 20 KEGG enrichment results, with pathway entries ranked in descending order based on their corresponding –log_10_(p-values).

**Figure 9:** – KEGG enrichment bubble plot (top 20 terms). KEGG pathway names are shown on the y-axis; p-values are displayed on the x-axis. Bubble size reflects the number of associated genes.

### Top 20 differentially expressed mRNAs:

Table 2 displays the top 20 mRNAs with differential expression.

**Table 2:** – Top 20 differentially expressed mRNAs in patients with CSF

Gene	Regulation	Fold chang*e*	p-value
SIRPB1	Up	1424.292887	8.76 × 10^-5^
OTOF	Up	749.2769265	0.033541446
CDC27	Up	372.4377459	0.000308112
FCGR1B	Up	293.4635502	0.002402462
HLA-DRB4	Up	288.483249	0.000897273
MEF2C	Up	279.7159676	7.28 × 10^-9^
ATG13	Up	241.9462662	0.001107754
EPB41	Up	173.0302956	0.004740905
ZNF45	Up	140.5873565	0.000840983
SLC39A9	Up	136.1707782	5.10 × 10^-5^
PBRM1	Down	0.007401108	6.99 × 10^-5^
P2RX5	Down	0.006490159	0.001694951
EPB41	Down	0.006205606	0.033667873
SRRM1	Down	0.006097372	1.07 × 10^-11^
ZNF445	Down	0.006009509	0.005438717
CCR3	Down	0.005964356	0.004797579
ALG13	Down	0.004730269	6.94 × 10^-7^
ABCA7	Down	0.004689293	0.019145994
KHSRP	Down	0.004116791	0.00809256
MAP4K4	Down	0.00314662	4.34 × 10^-6^

## Discussion

Transcriptome high-throughput sequencing in this study revealed that a total of 854 lncRNAs were differentially expressed, with 425 downregulated and 429 upregulated. Functional enrichment analysis identified 48 core GO entries. Co-expression and co-localization predictions highlighted metabolic processes and cellular metabolism under BP, and antioxidant activity, regulation of enzymatic activity, and catalytic activity under MF. Within the CC category, components such as cell junctions, extracellular matrix organelles, and intracellular organelles were correlated with the cytoplasm. KEGG analysis showed that differentially expressed lncRNAs were significantly enriched in pathways related to cardiovascular diseases, endocrine disorders, metabolic diseases, and neurodegenerative diseases.

LncRNAs are recognized for their involvement in both physiological and pathological processes, serving regulatory and structural functions in diverse biological activities, including genetic imprinting, epigenetic control, cellular proliferation, developmental processes, aging, and apoptosis, thus emerging as key modulators in numerous cardiovascular conditions.^12,32^ LncRNAs have been implicated in regulating CSF and its associated complications.^12,32,33^

In studies concerning DCAF4L1, the adjacent coding gene, which is a key component of the CUL4-DDB1 ubiquitin ligase complex, has been shown to influence tumor progression by modulating cell cycle checkpoints and apoptotic signaling pathways. A lncRNA potentially transcribed at this locus, referred to as lnc-DCAF4L1 (not yet officially annotated), is hypothesized to regulate immune inflammation. However, its precise mechanism requires experimental validation, such as through chromatin conformation capture (4C-seq).^34^

Notably, the classical lncRNA growth arrest-specific 5 (GAS5) plays distinct roles within disease networks. It regulates immune factors such as IL-10 via the competing endogenous RNA (ceRNA) mechanism in autoimmune diseases, modulates the insulin signaling pathway in metabolic disorders, and contributes to neuroinflammatory processes by influencing microglial activation in neurodegenerative conditions. This multifunctionality positions GAS5 as a central molecular hub for cross-disease research. While variations in the expression of protein-coding genes are acknowledged, further investigation is needed to elucidate their functional implications.^35,36^

Studies have shown that lncRNAs such as MALAT1 and NEAT1 play a role in promoting inflammatory responses in CSF through various molecular pathways.^4,12,18^ MALAT1 has been identified as a biomarker for predicting the no-reflow phenomenon during percutaneous coronary intervention (pPCI) and as a potential therapeutic target for CSF.^37,38^ Additionally, the correlation between lncRNA AF131217.1 levels and CSF has been investigated, suggesting a regulatory role for this lncRNA in CSF-induced inflammation through modulation of Krüppel-like factor 4 (KLF4).^38^ However, in the present study, MALAT1 and NEAT1 were not identified as having regulatory functions in patients with CSF.

Other differentially expressed lncRNAs identified in this study — including lnc-SLC46A2, lnc-RAB23, lnc-LIPI (Table 1), excluding lnc-DCAF4L1 and GAS5 — have not been previously reported in the literature. The relationship between these lncRNAs and CSF remains unclear and requires further investigation to be validated. Additional research is needed to clarify the specific mechanisms by which lncRNAs contribute to CSF and to explore their potential as therapeutic targets.

Among the 1,999 differentially expressed mRNAs, 990 were downregulated and 1,009 were upregulated. According to the GO clustering analysis of the top 10 entries, ranked by –log_10_(p-value), the main biological processes included response to viral infection, negative regulation of viral genome expression, type I interferon signaling pathway, mitochondrial depolarization, and activation of protease activity. The primary cellular components involved were the cytoplasm, nucleoplasm, Atg1/ULK1 kinase complex, nucleus, and phage assembly site. Molecular functions were predominantly related to protein binding, regulation of protein kinase activity, ubiquitin-protein transferase activity, and RNA binding. KEGG analysis showed that differentially expressed mRNAs were significantly enriched in the TLR signaling pathway and NF-κB signaling pathway.

The study by Coto et al.^37^ found that variation in NFKBIZ is an independent risk factor for early-onset CAD. Their research included 609 males with early-onset CAD and 423 healthy males in the control group. No significant inter-group differences were observed in the allele or genotype frequencies of NFKB1 rs28362491 (−94 delATTG) or NFKBIA rs8904; however, the frequency of the NFKBIZ rs3217713 deletion was significantly higher in the CAD group compared to controls.^37^ In our study, KEGG analysis indicated the NF-κB signaling pathway plays a role in the variation of mRNA expression, suggesting inflammation may be one of the underlying pathological mechanisms in CSF. However, research on the relationship between NFKBIZ and CSF is still in its early stages. The upregulated and downregulated mRNAs identified in this study (Table 2) have not been previously reported as being associated with CSF in the existing literature. Further research is needed to elucidate the specific mechanisms linking mRNAs to CSF.

Unlike previous research that often focused on individual lncRNAs or miRNAs, this study offers a more comprehensive perspective by integrating both lncRNA and mRNA expression profiles, as well as their interactions in the context of CSF. While earlier studies have suggested the involvement of lncRNAs such as MALAT1 in inflammatory responses and endothelial dysfunction, our findings specifically highlight the dysregulation of lncRNAs including ANRIL, MALAT1, and LINC00305 in patients with CSF, providing new insights into potential therapeutic targets and biomarkers.

### Limitations of study

The main limitation of this study is the small sample size, with only three individuals in each group. This limited number may not adequately capture biological variability, thereby reducing statistical power and potentially compromising the generalizability of the findings. Additionally, the study did not include other types of non-coding RNAs, such as microRNAs, which may also be relevant. These limitations suggest that future studies with larger and more diverse cohorts are essential to validate these results and to fully elucidate the mechanisms underlying CSF.

## Conclusion

In conclusion, this study confirms that lncRNAs — defined as transcripts longer than 200 base pairs — play crucial roles in CSF, with potential mechanisms primarily involving the modulation of inflammatory pathways and regulation of endothelial function. However, the upregulated and downregulated lncRNAs and mRNAs identified in this study have not been previously reported to be associated with CSF in literature. Further research is needed to clarify the specific mechanisms underlying the relationship between these transcripts and CSF.
